# Application of Invertebrate‐Derived DNA Barcoding (iDNA) in Blood Sucking Leeches From West Sumatra: A Discovery of Blue‐Eyed Litter Frog *Leptobrachium waysapuntiense*

**DOI:** 10.1002/ece3.72235

**Published:** 2025-10-01

**Authors:** Ardika Dani Irawan, Katsuyuki Eguchi

**Affiliations:** ^1^ Systematic Zoology Laboratory, Department of Biological Sciences, Graduate School of Science Tokyo Metropolitan University Tokyo Japan; ^2^ Department of Biology, Faculty of Mathematics and Natural Sciences Universitas Negeri Padang Padang West Sumatera Indonesia

**Keywords:** biodiversity, blue‐eyed litter frog, conservation, iDNA, noninvasive monitoring, terrestrial leeches

## Abstract

Indonesia is one of the world's most biodiversity‐rich countries, including a wide variety of vertebrate and plant species. However, assessing biodiversity in tropical rainforests remains challenging itself. The use of conventional tools has commonly been employed for monitoring and research purposes. Invertebrate‐derived DNA (iDNA), a subdiscipline of environmental DNA (eDNA), has emerged as a noninvasive tool that complements traditional methods for biodiversity assessment. It enables the detection of vertebrate species and the monitoring of their populations through molecular approaches. Utilizing abundant haematophagous leeches provides a promising approach to sample a broader range of host species within an area, as these leeches retain high‐quality host DNA in their guts for extended periods. Using Sanger sequencing with five primer sets (16Scp, 16Sed, 12S, ND2, and RepCOI) designed to target broad taxonomic groups, 272 *Haemadipsa* spp. samples were successfully amplified, resulting in the identification of 17 unique vertebrate hosts, including mammals, amphibians, and reptiles. Within our 16Sed results, we noted that the primer sets could capture a broader range of taxa than originally targeted, encompassing both mammals and reptiles, thereby enhancing species richness detection. Notably, we present evidence of the first iDNA‐based detection of the rare blue‐eyed litter frog, *Leptobrachium waysepuntiense* , from western Sumatra. Therefore, this study suggests that the use of haematophagous leeches represents a promising approach for biodiversity monitoring in Indonesia. This method offers a complementary strategy that can be integrated with existing practices to strengthen conservation efforts.

## Graphical abstract

This study explores the use of invertebrate‐derived DNA (iDNA) from haematophagous leeches to assess vertebrate biodiversity in the tropical rainforests of Indonesia. By applying Sanger sequencing with five primer sets, we detected 17 unique vertebrate taxa, including the rare *Leptobrachium waysepuntiense* , demonstrating the potential of iDNA as a complementary, noninvasive tool for biodiversity monitoring and conservation.

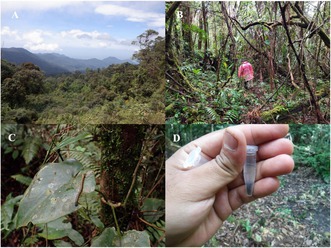

## 1 Introduction

Indonesia is a megadiverse country, comprising 10% of the world's total tropical rain forest and home to 10% of the world's mammal species, 16% of bird species, and 11% of plant species (Riswan and Yamada 2006). However, since 1950–2017, Indonesia had one of the highest rates of primary forest loss globally (Santoro et al. 2025). This has resulted in a significant decline in biodiversity. In many small vertebrate species (e.g., amphibians) with a body mass breakpoint of 0.039 kg, there is an elevated extinction risk (Ripple et al. 2017). Moreover, extinction risk in Indonesia is driven by multiple threats, with deforestation being among the most critical, alongside illegal wildlife poaching, wildlife trade, and climate change (Supriatna et al. 2020; Zhang et al. 2022).

To address this challenge, Indonesian researchers have conducted various field studies to monitor and prevent the decline in biodiversity (Haidir et al. 2024; Wibisono et al. 2011). Conventional methods such as camera trapping and direct observation have been shown to provide valuable data on species composition and the population size and dynamics of specific target species in a given area. These baseline data are indispensable for conservation planning (Wibisono et al. 2011; Inger and Iskandar 2005). Although camera trapping can be successfully manipulated to detect mammals of various sizes (Glen et al. 2013), the method is costly, prone to theft, and time‐consuming in terms of data analysis (Weiskopf et al. 2018). Furthermore, while it is generally expected that small mammal taxa have a high proportion of endemic species, most of the conventional survey methods, including camera trapping, tend to have low detection efficiency for small species (Fahmy et al. 2019).

As an alternative, iDNA (invertebrate‐derived DNA), a subdiscipline of environmental DNA metabarcoding (Schnell et al. 2018), has been introduced as a more sensitive, cost‐effective, and labor‐efficient approach to detecting small, ground‐dwelling vertebrate species (Calvignac‐Spencer et al. 2013). As the intestinal contents of blood‐sucking invertebrates may contain host DNA, the hosts can be identified by metabarcoding these contents. Various blood‐sucking invertebrates have been suggested as the targets for iDNA (Cutajar and Rowley 2020; Curler et al. 2015; Sawabe et al. 2010), with terrestrial leeches being considered particularly suitable (Fahmy et al. 2020; Schnell et al. 2018). Despite its potential, relatively few studies have applied iDNA to investigate amphibians (Ji et al. 2022). Many of these species in Sumatra and Indonesia are poorly known or under conservation threat. In Sumatra, while 66% of amphibian species are categorized as Least Concern, 14% are Data Deficient, and knowledge of their distribution and status is remains limited, especially for 52 endemic species (Arifin 2024).

One of the least understood aspects of leech ecology is their host range and feeding behavior. In particular, there is a lack of reports and research on their use of amphibians as hosts. The first description of an amphibian–leech interaction in Madagascar was reported by Rocha et al. (2012). A subsequent report has also found that the freshwater leech *Desserobdella picta* frequently becomes an ectoparasite of both larval and adult amphibians (Koprivnikar et al. 2012). In Hongkong, the interaction occurred between Asian common toad (*Duttaprhynus melanostictus*) and terrestrial leeches (*Tritetrabdella taiwana*) (Yuen and Nakano 2012), as well as between Asian painted frog (*Kaloula pulchra*) and *T. taiwana* (Nakano and Sung 2014). In India, *Duttaprhynus melanostictus* has also been found to be parasitized by the terrestrial leech *Haemadipsa sylvestris* (Mahapatra and Ghorai 2019). According to Morishima et al. (2020), anurans are known to be the most important hosts for leeches due to their overlapping habitats. However, to date, no studies have investigated whether terrestrial leeches in Indonesia parasitize anurans and other amphibians, likely due to the limited research focus on leeches and their host interactions in this region. Here, we report the use of iDNA to assess terrestrial vertebrate diversity and to document new geographic records of species. This study highlights the value of applying iDNA approaches, which can be further utilized to support biodiversity monitoring and conservation management in Indonesia.

## 2 Material and Methods

### 2.1 Sample Collection

**FIGURE 1:**
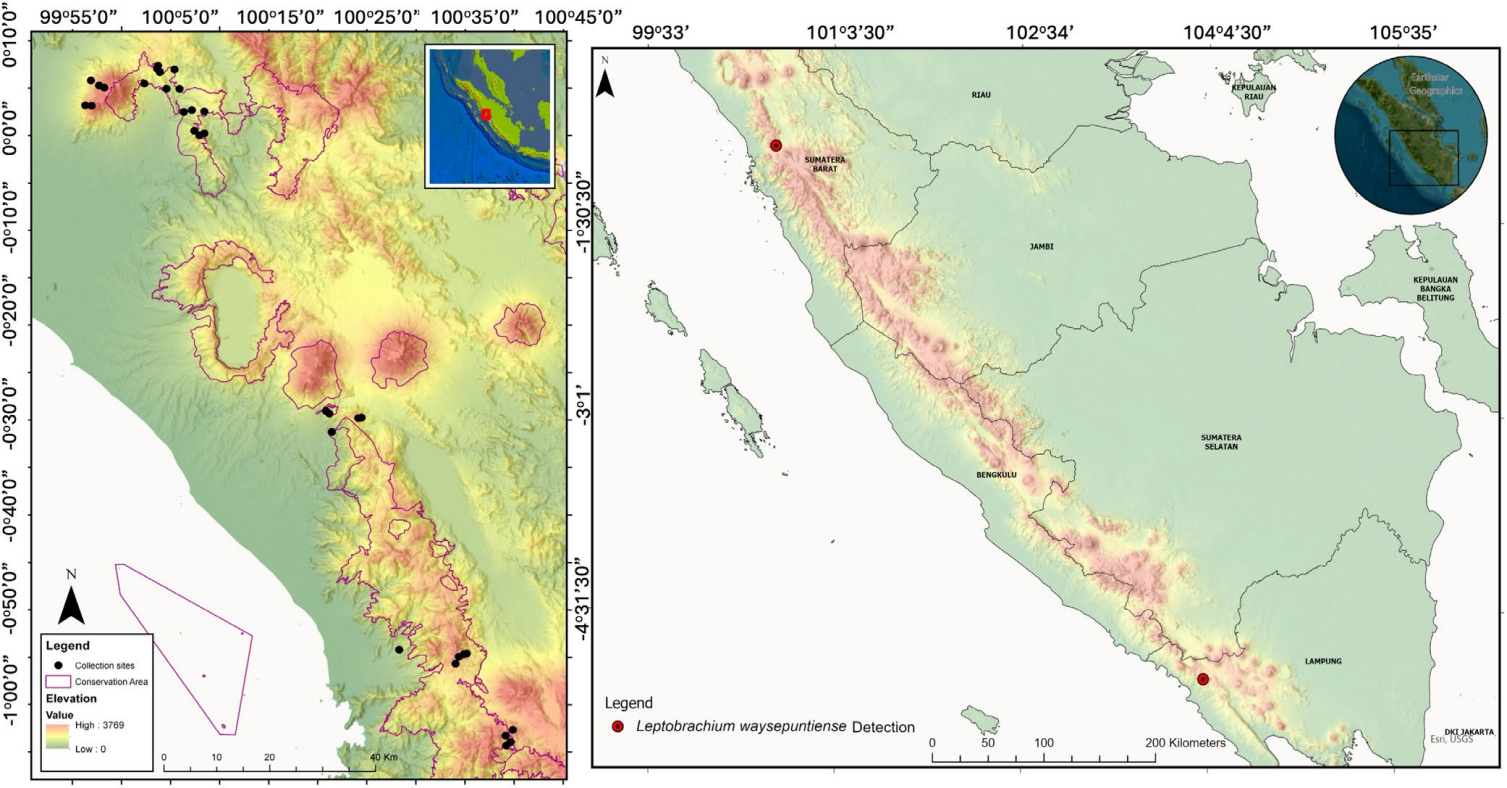
(A) Map of collection sites in West Sumatra. (B) The detection sites of blue‐eyed litter frog *Leptobrachium waysepuntiense*. Black box represented the first recorded the species from Kubu Perahu, Lampung province, Sourtern Sumatra (Hamidy and Matsui 2010).

**FIGURE 2:**
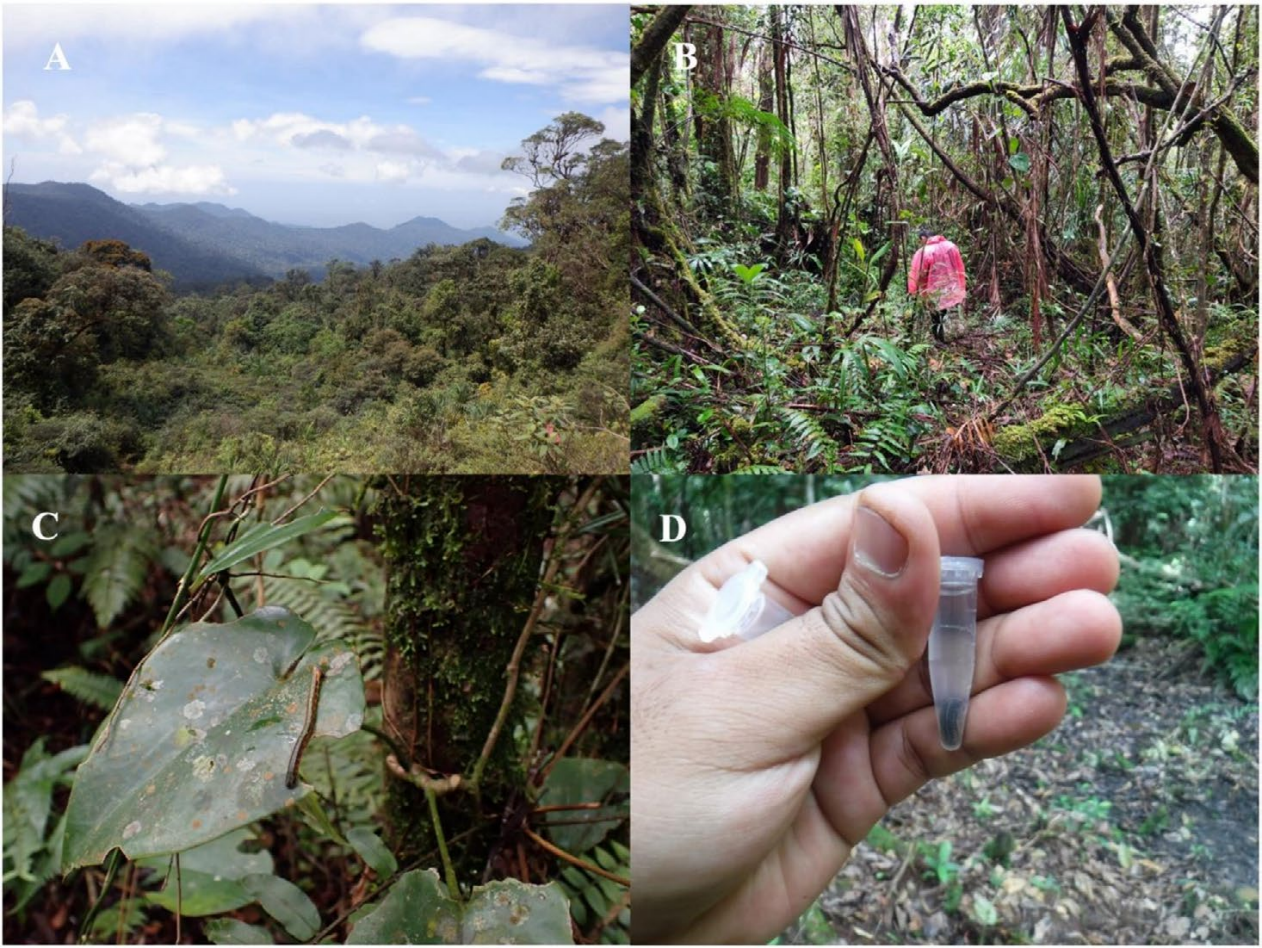
(A) General landscape of a forest in locality name. (B) Interior condition and trail route within Barisan Wild Reserve. (C, D) Terrestrial leeches (*Haemadipsa* spp.) collected in the forest by lead author.

A total of 272 *Haemadipsa* specimens collected from West Sumatra in 2020–2023 were used for the present iDNA analysis (Figures 1 and 2). The survey area covered a wide range of habitats and levels of human disturbance, from lowland forests (231 m a.s.l) to highland mountainous areas (2127 m a.s.l). In the Barisan Nature Reserve (BNR), 25 specimens were collected in areas along trails and streams in humid and wet forests (up to ~1000 m a.s.l); in the Tarusan Arau Ilir Nature Reserve (TAINR), 15 specimens from the areas with similar environmental conditions to those of the Barisan Nature Reserve; in the Biological Education and Research Forest (HPPB), which is located at the edge of the Barisan Nature Reserve, 17 specimens from alongside trails in low‐elevation secondary forests; in the Lembah Anai Nature Reserve (LANR), 70 specimens from primary and secondary forest; in the Malampah Alahan Panjang Wildlife Reserve (three sites) (MNR), 87 specimens from primary and secondary forests near agricultural zones; in Mt. Pasaman, 12 specimens from primary forests at the highest elevation (~1000 m); in Mt. Talamau, 48 specimens from primary forests located at the highest elevation (~1000 m). The elevation and geographical coordinates of each location were recorded using a Garmin GPSMAP 64 s SAE unit.

All leeches were found and collected by sight, either from the ground, low vegetation, or when they approached the collectors during the day. However, individuals observed feeding on collectors' blood were not collected. Night‐time sampling was generally avoided to ensure the safety of the team and minimize the risk of encountering large predators, even though the target leeches are also active at night.

### 2.2 Molecular Processing

DNA templates for iDNA were obtained from the gastrointestinal region of each leech. Approximately a quarter of the body, excluding the caudal sucker, was separated from the rest. One half (left or right) of the separated part was used for extraction, and it was finely chopped into small pieces on a sterile disposable dish in 500 μL of buffer TE (pH 8.0) and washed in the buffer. The other half was stored for future additional extraction. The pieces were then transferred to 105 μL of extraction buffer consisting of 100 μL of 10% Chelex‐TE solution and 5 μL of Qiagen proteinase K. The mixture was incubated at 56°C for approximately 24 h or more, depending on tissue lysis, and then heated at 99°C for 10 min to inactivate the proteinase K in the extraction buffer (Satria et al. 2015). The resulting DNA template was stored in a freezer at −25°C. See Supporting Information for details on DNA extraction.

Five gene regions were amplified and sequenced using the primer sets listed in Table 1. The abbreviations for these gene regions are as follows: 16Scp (272 specimens), 12S (249 specimens), 16Sed (213 specimens), RepCOI (92 specimens), and ND2 (*n* = 92 specimens). These regions have been used as standard gene markers for iDNA (Fahmy et al. 2020). Each polymerase chain reaction (PCR) contained 5 μL of 2× PCR Buffer, 2 μL of 2 mM dNTPs, 0.3 μL of forward primers, 0.3 μL of reverse primer, 1.7 μL of distilled water, 0.2 μL of KOD FX Neo, and 1 μL of DNA template. The amplification conditions were as follows: one cycle of 94°C for 2 min; 35 cycles of 98°C for 10 s, 54°C (RepCOI and ND2) or 50°C (12S, 16Scp, and 16Sed) for 30 s, 68°C for 45 s; and final annealing at 70°C for 2 min, followed by holding at 4°C indefinitely. Negative controls were included in all PCR runs to monitor potential contamination. The amplified products were confirmed using a 2% agarose gel. The ExoSAP process, which enzymatically cleans PCR product by removing excess deoxyribonucleotide triphosphates (dNTPs) and residual primer, was carried out by adding 2 μL of ExoSAP solution (1.0 μL of ExoSAP and 1.9 μL of distilled water) to an 8‐connected tube containing 5 μL of the PCR product. This was then incubated at 37°C for 4 min, followed by 80°C for 1 min.

**TABLE 1:** Primers used for the amplification of mitochondrial iDNA via the Sanger sequencing method.

Primer sets	Target genes (gene marker)	Nucleotides	Length (bp)	References
16SSCPF	Mammals (16S rRNA)	CGAGGGCTTTACTGTCTCTT	294	Caragiulo et al. (2014)
16SSCPR		CCTATTGTCGATATGGACTCT		
16SEDNAF1	Amphibian and Osteichthyes (16S rRNA)	AGACGAGAAGACCCYDTGGAGCTT	250	Vences et al. (2016)
16SEDNAR1		GATCCAACATCGAGGTCGTAA		
12SO	Vertebrate (12S rRNA)	CTGGGATTAGATACCCCACTAT	120	Poinar et al. (1998)
12SA		GTCGATTATAGGACAGGTTCCTCTA		
L5758	Avian (ND2)	GGNGGNTGAATRGGNYTNAAYCARAC	1059	Payne and Sorenson (2007)
H6313		ACTCTTRTTTAAGGCTTTGAAGGC		
REPCO1F	Reptile and amphibian (COI)	TNTTMTCAACNAACCACAAAGA	664	Nagy et al. (2012)
REPCO1R		ACTTCTGGRTGKCCAAARAATCA		

The products of the sequencing reaction were then purified and concentrated via ethanol precipitation with sodium acetate, after which Sanger sequencing was outsourced to FASMAC (https://fasmac.co.jp/en). The raw sequence data were assembled into contigs using ChromasPro version 2.1.10.1 (Technelysium Pty Ltd., Australia), a software with a graphical interface for visualizing and editing sequences.

The host species or taxa were determined by referring to the GenBank sequence database provided by the National Center for Biotechnology Information's (NCBI). BLASTn (NCBI's Nucleotide Basic Local Alignment Search Tool) was used to search for similar sequences in the publicly available DNA barcode libraries, specifically GenBank. An expectation value (E‐value) threshold of less than *e* ^−30^ to avoid low‐quality matches. A similarity threshold of 98% was used for species‐level identification (Weigand and Macher 2018). Due to the incompleteness of these libraries, coverage of Southeast Asian vertebrates, particularly amphibians and reptiles, is limited, and many endemic species are not yet represented; a second threshold of 90% similarity was also applied for family‐level identification (Porter and Hajibabaei 2018). All iDNA samples identified as *Homo sapiens* were excluded from the results and discussions.

### 2.3 *Leptobrachium* Phylogenetic Analyses

In order to identify the 16S sequences of the anuran genus *Leptobrachium* detected in this study, we selected reference sequences from GenBank that were both from the same genetic region and taxonomically relevant, including 20 anurans representing 13 sequences of Megophryidae, 3 sequences of Bufonidae, and 3 sequences of Rhacophoridae. We then used these sequences to reconstruct a phylogenetic tree (see Table 2). All sequences, including those generated in this study, were aligned using the MUSCLE algorithm (Madeira et al. 2024) in AliView (Larsson 2014) with the default setting. The phylogenetic tree was reconstructed using maximum likelihood estimation on the IQ‐TREE web server (http://iqtree.cibiv.univie.ac.at) (Trifinopoulos et al. 2016), with support values assessed via ultrafast bootstrap analysis (UFBoot) and the Shimodaira‐Hasegawa approximate likelihood ratio test (SH‐aLRT), both with 1000 replicates. The analyses were based on the GTR + F + G substitution model under the Bayesian Information Criterion (BIC).

**TABLE 2:** Samples of *Leptobrachium* species and related voucher specimens from GenBank data used for phylogenetic analyses in this study, along with location information, accession numbers, and references.

Species	Voucher Specimen/isolate	Locality	Genbank acc. no	References
*Leptobrachium lumadorum*	KU 314148	Philippines	GQ995549	Brown et al. (2009)
*Leptobrachium tagbanorum*	KU 309461	Philippines	GQ995551	Brown et al. (2009)
*Leptobrachium gunungense*	KUHE: 39377	Malaysia: Sabah	AB646405	Hamidy et al. (2011)
*Leptobrachium gunungense*	SP 3825a	Malaysia: Sabah	AB530404	Matsui et al. (2010)
*Leptobrachium montanum*	KUHE: 42811	Indonesia: South Kalimantan	AB646369	Hamidy et al. (2011)
*Leptobrachium montanum*	KUHE: UN larva	Indonesia: South Kalimantan	AB646370	Hamidy et al. (2011)
*Leptobrachium waysepuntiense*	UTA A53689	Indonesia: Jambi	AB530402	Hamidy and Matsui (2010)
*Leptobrachium* sp.	MZB: Amp:11313	Indonesia: North Sumatra	AB646388	Hamidy et al. (2011)
16SED‐16_BNR		Indonesia: West Sumatra		This study
*Leptobrachium waysepuntiense*	MZB Amp 15862	Indonesia: Lampung	AB530401	Hamidy and Matsui (2010)
*Megophrys nasuta*	ZCAK SEA0001	NA	LC640606	Kambayashi et al. (2022)
*Megophrys nasuta*	ZCYK Bcar1	NA	LC640472	Kambayashi et al. (2022)
*Megophrys monticola*		India	MH647510	Mahony et al. (2018)
*Megophrys parallela*	RMAS 022	Indonesia: West Sumatra	KY679898	Munir et al. (2018)
*Duttaphrynus melanostictus*	USNM594905	Myanmar	MT609692	Mulcahy et al. unpublished
*Duttaphrynus melanostictus*	USNM594906	Myanmar	MT609693	Mulcahy et al. unpublished
*Duttaphrynus melanostictus*	USNM594904	Myanmar	MT609691	Mulcahy et al. unpublished
*Rhacophorus poecilonotus*	ENS 19409 (UTA)	Indonesia: Aceh	KY886348	O'Connell unpublished
*Rhacophorus poecilonotus*	ENS 17629 (UTA)	Indonesia: Dempo	KY886345	O'Connell unpublished
*Rhacophorus poecilonotus*	ENS 17576 (UTA)	Indonesia: South Sumatra	KY886342	O'Connell unpublished

Tree nodes were considered “well supported” if UFBoot was 95% or higher (Minh et al. 2013) and SH‐aLRT was 80% or higher (Guindon et al. 2010). Uncorrected pairwise genetic distance (P‐distance) and Kimura two‐parameter (K2P) distances were calculated using MEGA 11 (Tamura et al. 2021).

## 3 Result

### 3.1 iDNA Assessment in West Sumatra

Each of the five gene markers was successfully amplified and sequenced, as summarized in Table 3. A total of 17 distinct vertebrate species were identified, comprising 15 mammals, one amphibian, and one reptile. These species belong to 14 families across six orders. Identifications that were considered to be geographically implausible were excluded based on the International Union for Conservation of Nature (IUCN) species distribution data (www.iucnredlist.org). Additionally, sequences from 16 leech specimens could only be assigned to seven different families and could not be confidently identified at the species level.

**TABLE 3:** The ID and label information of *Haemadipsa* specimens in which host DNA was detected by iDNA analysis, and the taxonomic names of the detected hosts (identification criteria are described in Section 2). The information on voucher or specimen code, collection locality, and GenBank accession number is indicated.

Specimen ID	Longitude	Latitude	Altitude	Locality	Habitat	Gene marker	Host			Accession no.
							Family	Genus	Species	
M3C24	100.12347	0.00864	897	MAPWR	Sf	16Scp	Suidae	Sus	*Sus scrofa*	PV800265
						12S	Suidae	Sus	*Undetermined*	PV765759
						16Sed	Suidae	Sus	*Sus scrofa*	PV801110
						RepCOI	Suidae	Sus	*Sus scrofa*	PV760979
						ND2	Suidae	Sus	*Undetermined*	PV855211
M3C38	100.13192	0.00083	972	MAPWR	Sf	16Sed	Felidae	Panthera	*Panthera tigris sumatraensis*	PV801111
M3C36	100.13192	0.00083	972	MAPWR	Sf	16Scp	Cervidae	Muntiacus	*Muntiacus muntjak*	PV800250
						12S	Cervidae	Muntiacus	*Muntiacus muntjak*	PV765760
M3C35	100.13192	0.00083	972	MAPWR	Sf	16Scp	Ursidae	Helarctos	*Helarctos malayanus*	PV800249
						12S	Ursidae	Helarctos	*Undetermined*	PV765761
						16Sed	Ursidae	Helarctos	*Helarctos malayanus*	PV801112
M3C28	100.13192	0.00083	972	MAPWR	Sf	16Scp	Cervidae	Muntiacus	*Muntiacus muntjak*	PV800243
						12S	Cervidae	Muntiacus	*Muntiacus muntjak*	PV765762
						16Sed	Cervidae	Muntiacus	*Muntiacus muntjak*	PV801113
M3C29	100.13192	0.00083	972	MAPWR	Sf	16Scp	Cervidae	Muntiacus	*Undetermined*	PV800241
						12S	Cervidae	Muntiacus	*Muntiacus muntjak*	PV765763
						16Sed	Cervidae	Muntiacus	*Muntiacus muntjak*	PV801114
B1C18	100.58597	−0.90995	1275	BNR	Pf	12S	Cervidae	Rusa	*Rusa unicolor*	PV765764
						16Sed	Cervidae	Rusa	*Rusa unicolor*	PV801115
B1C25	100.58597	−0.90995	1275	BNR	Pf	12S	Cervidae	Rusa	*Rusa unicolor*	PV765765
B1C43	100.58107	−0.91127	1485	BNR	Pf	12S	Cervidae	Muntiacus	*Muntiacus muntjak*	PV765766
B1C47	100.58107	−0.91127	1485	BNR	Pf	12S	Megophrydae	Leptobrachium	*Undetermined*	PV765767
						16Sed	Megophrydae	Leptobrachium	*Leptobrachium wayseputiense*	PV801116
T1C5	100.66429	−1.04422	1779	TAINR	Pf	16Scp	Bovidae	Bos	*Bos taurus*	PV800262
T1C33	100.65196	−1.05459	1778	TAINR	Pf	16Scp	Felidae	Catopuma	*Catopuma temminckii*	PV800258
						12S	Felidae	Catopuma	*Catopuma temminckii*	PV765768
						16Sed	Felidae	Catopuma	*Catopuma temminckii*	PV801109
T1C47	100.65991	−1.06608	1479	TAINR	Pf	16Sed	Tapiridae	Tapir	*Undetermined*	PV801108
T1C59	100.65991	−1.06608	1479	TAINR	Pf	16Scp	Bovidae	Bos	*Undetermined*	PV800254
T1C64	100.65991	−1.06608	1479	TAINR	Pf	16Scp	Bovidae	Bos	*Bos taurus*	PV800252
T1C65	100.65991	−1.06608	1479	TAINR	Pf	16Scp	Bovidae	Bos	*Bos taurus*	PV800251
						12S	Hystricidae	Hystrix	*Hystrix brachyura*	PV765769
H1C31	100.4716	−0.90328	325	HPPB	Pf	12S	Erinaceidae	Echinosorex	*Undetermined*	PV765770
A2C18	100.34711	−0.48408	580	LANR	Sf	16Scp	Cervidae	Muntiacus	*Muntiacus muntjak*	PV800248
						12S	Cervidae	Muntiacus	*Muntiacus muntjak*	PV765771
A2C19	100.34711	−0.48408	580	LANR	Sf	16Scp	Cervidae	Muntiacus	*Muntiacus muntjak*	PV800247
						12S	Cervidae	Muntiacus	*Muntiacus muntjak*	PV765772
A2C20	100.34711	−0.48408	580	LANR	Sf	12S	Cervidae	Rusa	*Undetermined*	PV765773
A2C40	100.35286	−0.48864	681	LANR	Pf	16Scp	Suidae	Sus	*Sus barbatus*	PV800246
						12S	Suidae	Sus	*Sus barbatus*	PV765774
A1C39	100.34711	−0.48408	580	LANR	Sf	16Scp	Cervidae	Muntiacus	*Muntiacus muntjak*	PV800241
						12S	Cervidae	Muntiacus	*Muntiacus muntjak*	PV765775
A2C26	100.35286	−0.48864	681	LANR	Pf	16Scp	Cervidae	Muntiacus	*Muntiacus muntjak*	PV800244
						12S	Cervidae	Muntiacus	*Muntiacus muntjak*	PV765776
A1C65	100.35286	−0.48864	681	LANR	Pf	16Scp	Viverridae	Paguma	*Paguma larvata*	PV800242
						12S	Viverridae	Paguma	*Undetermined*	PV765777
M1C19	100.06468	0.11162	1018	MAPWR	Sf	16Sed	Cervidae	Muntiacus	*Muntiacus muntjak*	PV801107
M1C31	100.06031	0.11785	1029	MAPWR	Sf	16Sed	Ursidae	Helarctos	*Helarctos malayanus*	PV801118
M1C60	100.08973	0.11624	875	MAPWR	Sf	16Sed	Cervidae	Rusa	*Rusa unicolor*	PV801119
M1C65	100.08973	0.11624	875	MAPWR	Sf	16Sed	Cervidae	Neofelis	*Undetermined*	PV801120
M1C87	100.09828	0.08228	587	MAPWR	Sf	16Sed	Felidae	Panthera	*Undetermined*	PV801121
M1C90	100.09828	0.08228	587	MAPWR	Sf	16Sed	Felidae	Panthera	*Panthera tigris sumatrae*	PV801122
M2C2	100.10565	0.04174	402	MAPWR	Sf	16Scp	Felidae	Panthera	*Panthera tigris sumatraensis*	PV800240
						16Sed	Felidae	Panthera	*Panthera tigris sumatrae*	PV801117
M2C8	100.10565	0.04174	402	MAPWR	Sf	16Scp	Tapiridae	Tapirus	*Tapirus indicus*	PV800239
						16Sed	Tapiridae	Tapirus	*Tapirus indicus*	PV801123
M2C33	100.11919	0.045	713	MAPWR	Sf	16Scp	Felidae	Panthera	*Panthera tigris sumatraensis*	PV800238
M2C41	100.11919	0.045	713	MAPWR	Sf	16Sed	Felidae	Prionailurus	*Prionailurus bengalensis*	PV801124
M2C42	100.11919	0.045	713	MAPWR	Sf	16Scp	Felidae	Panthera	*Panthera tigris sumatraensis*	PV800264
M2C43	100.11919	0.045	713	MAPWR	Sf	16Scp	Felidae	Panthera	*Panthera tigris sumatraensis*	PV800263
						12S	Felidae	Panthera	*Undetermined*	PV765778
						16Sed	Felidae	Panthera	*Panthera tigris sumatraensis*	PV801125
M2C55	100.14033	0.04264	775	MAPWR	Sf	16Sed	Suidae	Sus	*Sus scrofa*	PV801126
A2C37	100.35286	−0.48864	681	LANR	Pf	12S	Varanidae	Varanus	*Varanus rudicollis*	PV765779
M3C40	100.13192	0.00083	972	MAPWR	Sf	16Sed	Felidae	Neofelis	*Undetermined*	PV801128
A1C56	100.352861	−0.48863	681	LANR	Pf	16Sed	Cervidae	Muntiacus	*Muntiacus muntjak*	PV801129
A2C46	100.35286	−0.48864	681	LANR	Pf	16Sed	Tragulidae	Tragulus	*Tragulus javanicus*	PV801130
A2C105	100.356944	−0.52097	329	LANR	Sf	16Sed	Suidae	Sus	*Sus scrofa*	PV801131
B1C50	100.58107	−0.91127	1485	BNR	Pf	16Sed	Tapiridae	Tapirus	*Tapirus indicus*	PV801132
B1C62	100.58107	−0.91127	1485	BNR	Pf	12S	Tapiridae	Tapirus	*Undetermined*	PV765780
M1C36	100.03842	0.09167	1300	MAPWR	Pf	16Sed	Ursidae	Helarctos	*Helarctos malayanus*	PV801133
M1C43	100.03842	0.09167	1300	MAPWR	Pf	16Sed	Cervidae	Muntiacus	*Muntiacus muntjak*	PV801134
M1C46	100.03842	0.09167	1300	MAPWR	Pf	16Sed	Tapiridae	Tapirus	*Undetermined*	PV801135
M1C10	100.06188	0.12321	950	MAPWR	Sf	16Scp	Sciuridae	Lariscus	*Undetermined*	PV800261
						12S	Sciuridae	Lariscus	*Undetermined*	PV765781
M1C15	100.06468	0.11162	1018	MAPWR	Sf	12S	Ursidae	Helarctos	*Undetermined*	PV765782
						16Scp	Ursidae	Helarctos	*Helarctos malayanus*	PV800260
M2C27	100.10564	0.04173	402	MAPWR	Sf	16Scp	Ursidae	Helarctos	*Helarctos malayanus*	PV800259
T2C51	99.97066	0.08453	2127	MTT	Pf	12S	Mustelidae	Arctonyx	*Undetermined*	PV765783
T2C58	99.9706564	0.0845288	2127	MTT	Pf	12S	Mustelidae	Arctonyx	*Undetermined*	PV765784
T2C42	99.97066	0.08453	2127	MTT	Pf	16Scp	Bovidae	Capricornis	*Capricornis sumatraensis*	PV800257
						12S	Bovidae	Capricornis	*Capricornis sumatraensis*	PV765785
T2C20	99.96167	0.08831	1733	MTT	Pf	16Scp	Viverridae	Paguma	*Paguma larvata*	PV800255
						12S	Viverridae	Paguma	*Paguma larvata*	PV765786
T2C28	99.96167	0.08831	1733	MTT	Pf	16Scp	Felidae	Catopuma	*Catopuma temminckii*	PV800255
						12S	Felidae	Catopuma	*Catopuma temminckii*	PV765787
PSMC52	99.93839	0.05336	1249	MTP	Pf	16Scp	Viverridae	Paguma	*Paguma larvata*	PV800253

Abbreviations: BNR, Barisan Nature Reserve; LANR, Lembah Anai Nature Reserve; MAPWR, Malampah Alahan Panjang Wildlife Reserve; Pf, Primary Forest; Sf, Secondary Forest; TAINR, Tarusan Arau Ilir Nature Reserve.

The iDNA for 16Scp detected seven families belonging to three orders (Carnivora, Artiodactyla, and Rodentia), with an overall detection rate of 9.92% (27 out of 272 samples). The most frequently encountered family was Cervidae (2.57%), followed by Felidae (2.20%). Furthermore, *Bos taurus* , domestic cattle species, was also detected in the protected zone of TAINR.

The iDNA for 16Sed detected eight families belonging to five orders (Carnivora, Artiodactyla, Perissodactyla, Anura, and Squamata), with an overall detection rate of 13.14% (28 out of 213). The highest detection rate was for Felidae (4.23%), followed by Cervidae (3.29%). At the species level, the highest detection rate was for *Muntiacus muntjak* (barking deer) at 2.34%, followed by *Panthera tigris sumatrae* (Sumatran tiger) at 1.87%. Additionally, the iDNA also detected *Leptobrachium waysepuntiense* (blue‐eyed litter frog), a rare endemic species that had previously only been recorded in southwestern Sumatra (Hamidy and Matsui 2010).

The iDNA for 12S detected 13 families belonging to seven orders (Artiodactyla, Carnivora, Eulipotyphla, Perissodactyla, Rodentia, Anura, and Squamata), with an overall detection rate of 11.64% (29 out of 249). This gene marker detected the highest number of families. The most frequently detected family was Cervidae (4.41%), followed by Felidae (1.20%). At the species level, *Muntiacus muntjak* (barking deer) was the most frequently detected (3.21%), surpassing all other species. Furthermore, a rare species, *Capricornis sumatraensis* (Sumatran serow), was only detected in one leech sample collected from Mt. Talamau at 2127 m a.s.l. This is the nominotypical subspecies that has been found to be entirely restricted to the volcanic mountain chain of the Bukit Barisan mountains in Sumatra (Phan et al. 2020). It typically occurs between 200 and 3000 m a.s.l. and inhabits both primary and secondary forests (Santiapillai 1997).

Neither the ND2 nor the RepCOI markers detected any nonhuman iDNA targets, except for a single detection of *Sus scrofa* (wild boar). Despite the presence of several ground‐dwelling birds inhabiting the Sumatran rainforests, ND2 was unsuccessful in detecting the target avian species. RepCOI, which was specifically designed to target amphibians and reptiles, was also unsuccessful in detecting these groups, which were detected with 16Sed and 12S.

Overall, this study detected at least four of the six wild cat species recorded in Sumatra, that is, *Panthera tigris* , *Catopuma temminckii* , *Prionailurus bengalensis* , and *Neofelis nebulosa*. All of these species are globally threatened (Goodrich et al. 2022; Hearn et al. 2015; Petersen et al. 2021).

Conversely, there were only two species‐level determinations that were geographically implausible: *Dendropsophus seniculus* from Brazil (PID 100%, E‐value 0.022) and *Phyllodactylus lanei rupinus* from Mexico (PID 100%, E‐value 0.015). As negative controls included throughout the experiment showed no amplification, and the Systematic Zoology Laboratory has not conducted any analyses of vertebrates for at least the past 15 years, it is not thought that these implausible detections were due to contamination within the laboratory.

### 3.2 *Leptobrachium* Phylogenetic Relationship

**FIGURE 3:**
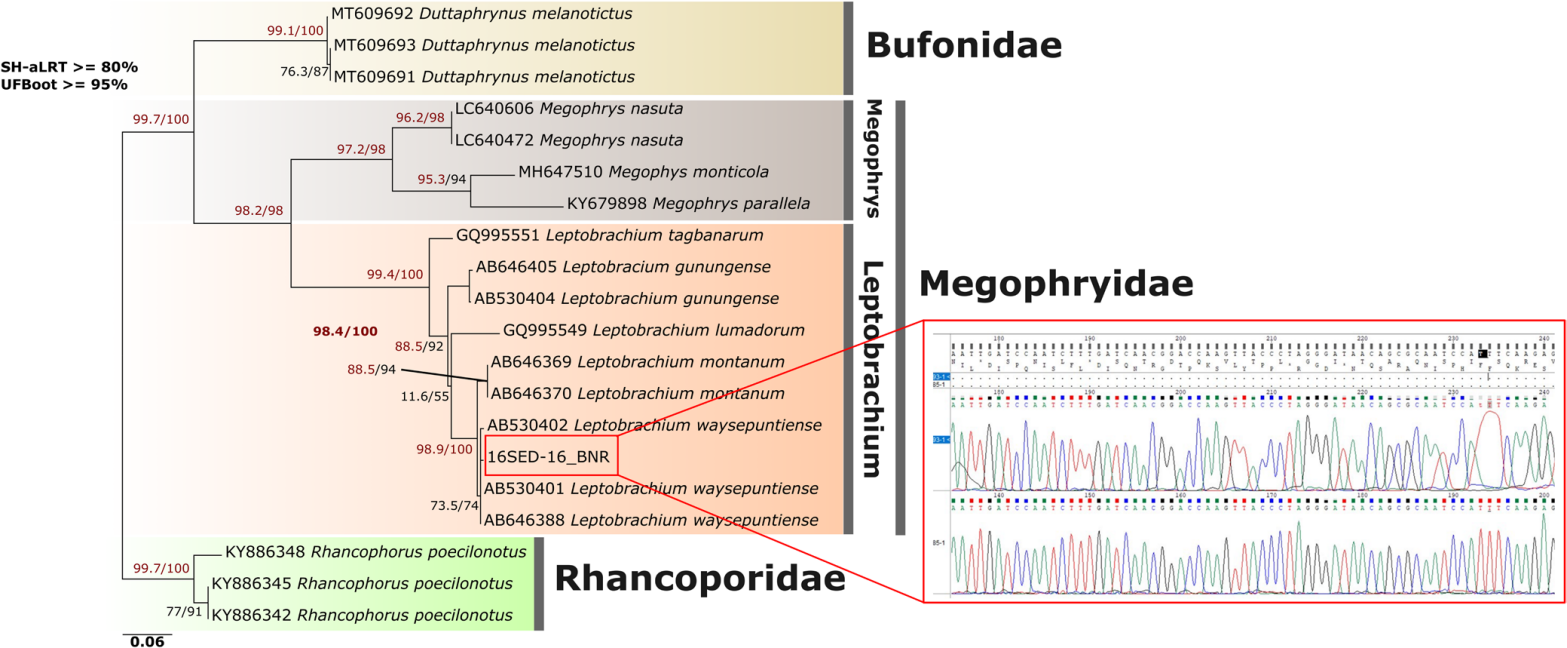
Maximum Likelihood phylogenetic tree of the Megophryidae group. The red box highlights the sequence detected by the present iDNA, supported by a clear chromatogram of the DNA sequence.

The Maximum Likelihood (ML) analysis successfully reconstructed the phylogeny using 10 sequences, including both public NCBI data and our newly generated sequence. The analysis produced a topology with a log‐likelihood (ln L) of −6707.693. Overall, the relationships among taxa were strongly supported, as indicated by high nodal support values. In our dataset, the phylogenetic analysis (Figure 3) placed our sequence as a singleton within the *Leptobrachium waysaputiense* clade, forming a short branch. Pairwise genetic distance analyses (p‐distance and K2P; Table 4) showed no divergence between our sequence and *L. waysaputiense*, further supporting this identification. The analysis also recovered a close sister relationship between *L. montanum* and *L. waysaputiense* (SH‐aLRT = 98.9; UFBoot = 100). The monophyly of *L. montanum* received moderate to strong support (SH‐aLRT = 88.5; UFBoot = 94), whereas that of *L. waysaputiense* was weakly supported (SH‐aLRT = 73.5; UFBoot = 74).

**TABLE 4:** Uncorrected genetic distance (P‐distance) and Kimura two‐parameter (K2P) within several anuran groups for the 16S rRNA fragment. The bold letters within the red box indicate no genetic distance between the respective taxa groups. The abbreviations for taxa are as follows: A = *Leptobrachium lumadorum* , B = *Leptobrachium tagbonarum*, C1–2 = *Leptobrachium gunungense* , D1–2 = *Leptobrachium montanum* , E1–3 = *Leptobrachium waysepuntiense* , 16Sed = This study, F1–3 = *Megophrys nasuta* , G = *Megophrys monticola* , H = *Megophrys parallela* , I1–3 = *Duttaphrynus melanostictus* , J1–3 = *Rhacophorus poecilonotus* .

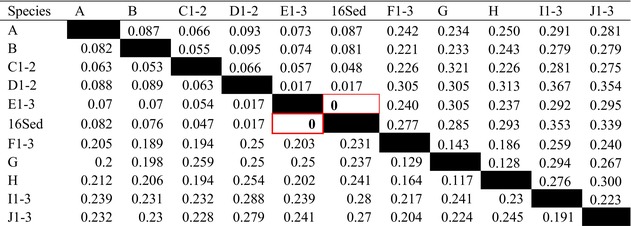

## 4 Discussion

### 4.1 The Host Animal Specificity of *Haemadipsa*

From 2020 and 2023, we collected a considerable number of terrestrial leeches (*Haemadipsa* spp.) annually. During fieldwork, we surveyed various physical conditions and found that *Haemadipsa* leeches favored moist and wet forests, resulting in high abundance during surveys, as observed in Barisan Nature Reserve, for instance.

This study indicated that the use of iDNA is a promising tool for detecting and monitoring rare vertebrate species and small amphibian species in Barisan Nature Reserve (BNR), one of Indonesia's most important conservation areas. These results are consistent with those of previous studies (Fahmy et al. 2023; Weiskopf et al. 2018; Wilting et al. 2022). Moreover, genetic markers worked relatively well in detecting vertebrates; for instance, although the 16Sed locus was originally developed for amphibians and osteichthyans, it was effective in identifying other animal groups, even rare species of mammals. In addition, the 16Scp and 12S loci were also contributed to detecting the rare and elusive mammals.

**FIGURE 4:**
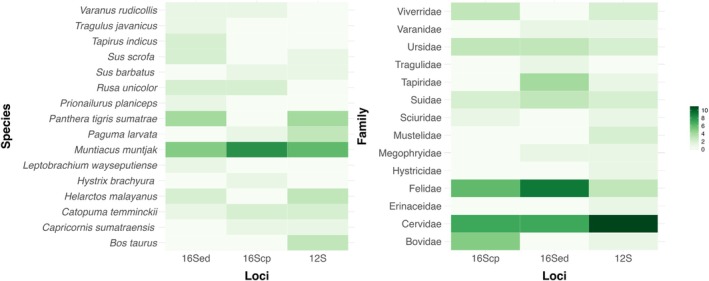
Proportion of host detections at different taxonomic levels using iDNA analyses across three loci. Values represent the number of host detections per individual bloodmeal. Color version available online.

While, although vertebrates were not found in all the extracted samples, iDNA currently highlights the potential of leech‐derived iDNA as a complementary and effective tool for monitoring rare and elusive threatened species, for example, critically endangered species (CR) Sumatra tiger *Panthera tigris sumatrae* (*n* = 5; MAPWR), endangered species (EN) Malayan tapir *Tapirus indicus* (*n* = 2; BNR, MAPWR), and Vulnerable species (VU) Sumatran serow *Capricornis sumatraensis* (*n* = 1; TAINR) (Figure 4, Figure S1). The ability of iDNA to detect terrestrial vertebrates of conservation importance is comparable to that demonstrated in a recent study of the rare rabbit species, the Annamite striped rabbit *Nesolagus timminsi* , in Vietnam (Nguyen et al. 2021).

Although the detection rate per leech appears low, this outcome is not unexpected given that we sequenced each leech individually. Many previous iDNA studies have employed pooled leech extractions (Drinkwater et al. 2020; Siddall et al. 2019; Schnell et al. 2018), which naturally increase the probability of host detection. Our individual‐based approach therefore provides comparable detection efficiency while also allowing for a more precise estimation of host occurrence at the level of single leeches (Weiskopf et al. 2018).

Three vertebrate groups, comprising birds, nonhuman primates, and fish, were not detected in our dataset. This is noteworthy because previous leech iDNA studies in Southeast Asia have reported these taxa. For instance, primates, particularly rhesus macaques (*Macaca mulatta*), were frequently detected in Bangladesh (Weiskopf et al. 2018), and avian taxa, although at a low rate, were also recovered from *Haemadipsa sylvestris* bloodmeals in Laos/Vietnam (Schnell et al. 2018). In this study, the absence of primates may be due to local ecological conditions, with leech energy demands primarily fulfilled by large mammals present at the survey site. For fish, it is plausible that nondetection relates to sampling bias. According to the lead author's observations during the field survey, *Haemadipsa* leeches cannot swim but are capable of crawling out after dropping into water. This suggests a potential capability for seeking out hosts in aquatic or semi‐aquatic habitats. Additionally, most samples in this study were collected along ridges and trails at some distance from riverbanks. Consequently, the opportunity for fish‐feeding events may have been very limited in the present study.

In this study, we found that Felidae and Cervidae were detected more often, which may reflect a preference by *Haemadipsa* leeches for hosts that provide more nutritional resources to the leeches rather than that of other mammalian families. It is similar to the previous study by Morishima et al. (2020), where *Cervus nippon* (Cervidae) had high detection in *Haemadipsa* bloodmeals in Japan. Similarly, in Borneo, Cervidae is the highest family detected from bloodmeals (Drinkwater et al. 2020). Then, we taught an important point about our finding is that the Felidae could be an additional preferred host, providing substantial energy for leeches, alongside Cervidae. This may represent the first documented occurrence of the leeches‐host relationship. When compared to previous findings from Malaysia, where Felidae detections were relatively low despite both countries having similar habitat and species composition (Abrams et al. 2019).

The relative densities of the major mammalian families in the BNR are poorly understood. However, trends reported by Wibisono (2021) across Sumatra suggest that the population of ungulates represented by sambar deer, barking deer, and wild boar were generally healthy, based on naïve occupancy estimates (0.98 ± 0.01, 0.90 ± 0.03, 0.89 ± 0.03, respectively). We suggest that the pattern in our study may partly reflect the host preference of *Haemadipsa*, at least in part, because the density of the medium‐to‐large‐sized predatory Felidae is likely much lower than that of the herbivorous Cervidae. We are now reexamining the species‐level taxonomy of *Haemadipsa* using an integrative approach of morphological observation and molecular phylogenetic analysis. This is because, if host and microenvironment preferences differ among *Haemadipsa* species, this could potentially introduce a bias that affects the results of iDNA.

Domestic cattle are frequently detected via iDNA within the protected zone of TAINR, which may not represent laboratory contamination. Similar findings have been reported by Ji et al. (2022) in China, where the domestic species (cattle, goat, and sheep) were also the most frequently detected in protected areas. This finding raises important concerns, particularly given the high incidence of human–wildlife conflict in West Sumatra. Considering this, the use of leech‐derived DNA offers effectiveness for monitoring protected areas (Ji et al. 2022). To our knowledge, according to local news reports, the rate of tiger–human conflict in West Sumatra remains high. Throughout 2024, at least 24 cases were reported, including livestock depredation and sightings in residential or agricultural areas. Many of these cases have resulted in tiger mortality. Notably, West Sumatra had one of the highest mortality rates (0.26 tigers per incident) among all mainland Sumatra provinces (Kartika 2017). Therefore, this evidence could be used to inform conservation authorities, such as the Conservation of Natural Resources (BKSDA), as well as local community initiatives, by identifying areas at high risk of livestock grazing within protected zones. This, in turn, would target interventions, such as improved monitoring and community‐based management, to reduce anthropogenic activities at forest edges and mitigate human–wildlife conflict.

### 4.2 The Usefulness of iDNA in Amphibian Surveys, a Case of *Leptobrachium waysaputiense* Detected by iDNA

Anurans have been identified as the primary hosts for *H. japonica* in regions where large mammals, such as sika deer, are absent (Morishima et al. 2020). Prior to this study, haemadipsid‐amphibian parasitism had been described for a wide variety of species in several studies (Merila and Sterner 2002; Nakano and Sung 2014; Raffel et al. 2006; Rocha et al. 2012). As discussed below, this study detected one frog species, *Leptobrachium waysaputiense*, whose distribution and habitat preference are poorly understood. In general, amphibians dwell in moist forests and areas near small streams. Consequently, amphibian habitats often overlap with leech habitats, providing opportunities for leeches to parasitize amphibians (Morishima et al. 2020).

Most species in the family Megophryidae are known to be effective at camouflaging themselves in their environment, often displaying body coloration that resembles leaf litter. Due to this effective camouflage, the frogs were rarely observed directly during the field survey. In terms of revealing a local amphibian fauna, iDNA targeting leeches collected near water could be an effective complementary method to traditional approaches such as direct observation and specimen capture.

This study detected a megophryid frog species in the blood meal of a single *Haemadipsa* individual collected from BNR (Table 3). iDNA with 12S showed moderate similarity (96.48%) with *Leptobrachium montanum* (isolate: KUHE 53f783; GenBank accession number: AB646386), with an E‐value of 6^−57^, while iDNA with 16Sed showed high similarity (99.29%) with *L. waysaputiense* (isolate: MZB Amp 11313; AB530401), with an E‐value of 5^−139^. Because 12S sequences only allowed identification at the family level, this study considered it as belonging to the Leptobrachium family. Moreover, we described more information for the 16S sequence.

*Leptobrachium waysaputiense* was described and named for the first time by Hamidy and Matsui (2010) based on specimens collected only from the type locality in West Lampung, which is approximately 600 km apart from BNR. Although at present the only single confirmed distribution record so far is from the type locality, the authors suggested that “ *L. waysepuntiense* is almost certainly distributed in western part of Jambi, as well, because a specimen of *Leptobrachium* from Jambi has a mtDNA haplotype very close to this species (Matsui et al. unpublished data).” Thus, this study provides further evidence to support the idea that this species has a relatively wide distribution, including West Sumatra. However, due to the lack of studies in recently surveyed areas, population estimates remain limited.

*L. waysepuntiense* is categorized as Least Concern (LC) by the IUCN (IUCN SSC Amphibian Specialist Group 2018). In fact, the impact of deforestation, combined with a few pieces of information recently, suggests that this species may become more threatened in the future. During our study, we observed a relatively high level of deforestation activity in BNR. Therefore, continuous amphibian surveys and reevaluation of status is also required for Sumatran species (Arifin 2024). The *Haemadipsa* specimen, from which *L. waysepuntiense* was detected, was collected from the humid forest floor, which was covered in foliage, at an elevation of 1485 m a.s.l. During the field survey period, the area experienced relatively high humidity (19.1 g/kg) and consistently low‐intensity precipitation (3.31 mm/day) (NASA LaRC POWER Project 2024). Hamidy and Matsui (2010) reported that the type series of *L. waysepuntiense* was collected in a primary forest at a higher elevation (691–852 m a.s.l.), approximately 250 m from a rocky stream. This suggests that the intensive survey of areas alongside streams in montane forests of Sumatra can reveal the geographic range of the species and provide further insight into its conservation status.

### 4.3 The Benefit of Multilocus and Potential Bias Detection

The use of multilocus Sanger sequencing in the present study provided valuable insights and enhanced the detection outcomes. On the other hand, as explained by Fahmy et al. (2019), the Sanger sequencing approach generally detects only a single blood meal per leech. Nevertheless, this approach is less sensitive and powerful than Next Generation Sequencing (NGS) (Schuster 2008), which can detect DNA from multiple species within a single invertebrate‐derived DNA sample (Hanya et al. 2019). Interestingly, the present study yielded more successful results than those of Fahmy et al. (2019), who, despite using the same Sanger sequencing approach, did not detect any threatened species. In contrast, our findings using Sanger sequencing were more consistent with those of Fahmy et al. (2020), who applied NGS and successfully detected threatened species.

Considering that each locus was originally designed to target specific taxonomic groups, the range of vertebrates detected is often restricted to those taxa. However, by applying a multilocus approach, these limitations can be mitigated, and detected more than one host DNA in some cases, we did not. For instance, although the 16Sed locus was originally developed for specific taxa (Table 1), in this study it also proved effective in detecting a broader taxonomic range, including mammals. This highlights the utility of combining multiple loci to improve detection coverage and reduce potential taxonomic bias in iDNA studies.

Although not included in analyses, human DNA was frequently detected in the blood meals. There are several possible explanations for this observation: (1) true human DNA, (2) mixed primate DNA in which human DNA is preferentially amplified, or (3) the absence of nonhuman primate DNA. Because this study did not employ a specific blocking primer design as used by Hanya et al. (2019), the detection rate of human DNA was relatively high. In addition, according to Schnell et al. (2015), *Haemadipsa* leeches are not ideal for detecting arboreal species; however, they may still feed on primates, as several primate colonies in West Sumatra such as pig‐tail macaque *Macaca nemestrina* and long‐tail macaque *Macaca fascicularis* are semi‐terrestrial and are often observed them playing and foraging on the forest floor. Referring to the fact that avian and osteichthyan species were not detected, we do not believe this was due to primer inefficiency on those prey taxa, as we used taxon‐specific generic primers (Fahmy et al. 2020). Instead, we conclude that this may reflect a feeding preference of the leeches toward hosts with larger body mass, and the relative distance of collection sites was far from the river.

Some leech host detections assigned to geographically implausible families are likely the result of noisy or short amplified nucleotide fragments. This explanation seems more plausible, as it may affect the accuracy of host identification, in addition to the possibility of closely related species not yet represented in the NCBI database, as noted by Fahmy et al. (2020).

### 4.4 Practical Applicability of iDNA in Indonesia

Due to limitations in the availability of DNA sequences and in the precision of the annotation to the sequences in the NCBI GenBank database, one of the 17 vertebrate species we detected did not have reference sequences available. So, the similarity of species in DNA‐based identification should sometimes be carefully reviewed in the context of species' geographic distribution. *Neofelis nebulosa* (clouded leopard), which was historically considered a single species, has been divided into two distinct species based on molecular and morphometric analyses (Christiansen 2008). Our iDNA result showed a strong match with *N. nebulosa* ; however, this species is known to inhabit only mainland Southeast Asia. In contrast, *N. diardi* (Sunda clouded leopard) is distributed across Sumatra and Borneo (Hearn et al. 2015). However, reference sequences annotated with the name of *N. diardi* were unavailable in the library. Therefore, in accordance with previous studies (Haidir et al. 2013, 2021; Sunarto et al. 2015), we suggest that our iDNA result initially identified as *N. nebulosa* should be reinterpreted as *N. diardi* .

Sample collection had been attempted previously in Sumatra; however, due to export permit issues, the samples could not be further analyzed (Weiskopf et al. 2018). Therefore, this study can be considered the first iDNA survey in Sumatra using Sanger sequencing, addressing the gap left by previous studies. By applying Sanger sequencing, which allows the library to be prepared easily with limited laboratory facilities (Schnell et al. 2012; Weiskopf et al. 2018; Fahmy et al. 2019), it should be noted that if the gut of a single leech contains blood from multiple host species, Sanger sequencing will not distinguish between the sequences from the different species without a cloning procedure. Even if PCR amplification succeeded, this will not allow us to detect the host species. This may explain the low detection rate beside the other variables, number of leeches analyzed, and methodology in this study. However, if iDNA is performed using next‐generation sequencing, as in the previous research (Fahmy et al. 2020; Srivathsan et al. 2023; Fernandes et al. 2023), the detection efficiency of the host species should increase. Furthermore, the final cost‐effectiveness and practical usefulness should also increase due to the higher sensitivity of NGS compared to Sanger sequencing (Deiner et al. 2017; Schuster 2008).

Assuming that iDNA is used in developing countries including Indonesia, the main issue will be the availability of the expensive equipment required to prepare the libraries for next‐generation sequencing. Conversely, the expensive equipment required to prepare the libraries for Sanger sequencing (without cloning) is limited to a thermal cycler for PCR amplification and labeling, as well as a freezer and a refrigerator for storing reagents. Therefore, it can be concluded that iDNA using Sanger sequencing is practical for conservation actions in developing countries.

To evaluate the effectiveness of iDNA, it is useful to compare the data obtained using iDNA with data obtained using conventional methods (e.g., visual observation and camera traps) in the same localities. In West Sumatra, where we are currently collecting *Haemadipsa* leeches in preparation for the large‐scale implementation of iDNA based on this study, intensive surveys using camera traps were previously conducted. Therefore, such a comparison will be conducted in a separate study.

## Author Contributions

**Ardika Dani Irawan:** conceptualization (equal), data curation (lead), formal analysis (lead), investigation (equal), methodology (equal), resources (equal), software (lead), visualization (lead), writing – original draft (lead), writing – review and editing (equal). **Katsuyuki Eguchi:** conceptualization (equal), data curation (equal), formal analysis (equal), funding acquisition (lead), investigation (equal), methodology (equal), project administration (lead), resources (equal), supervision (lead), validation (equal), visualization (equal), writing – original draft (equal), writing – review and editing (equal).

## Conflicts of Interest

The authors declare no conflicts of interest.

## Supporting information


**Figure S1:** ece372235‐sup‐0001‐FigureS1.docx.

## Data Availability

The data that support the findings of this study are openly available in Application of Invertebrate‐derived DNA barcoding at https://doi.org/10.6084/m9.figshare.30090979.v1.
