# Correction to Application of Invertebrate‐Derived DNA Barcoding (iDNA) in Blood‐Sucking Leeches From West Sumatra: A Discovery of Blue‐Eyed Litter Frog *Leptobrachium waysapuntiense*

**DOI:** 10.1002/ece3.72553

**Published:** 2025-11-20

Irawan, A. D., and Eguchi, K. (2025). Application of Invertebrate‐Derived DNA Barcoding (iDNA) in Blood‐Sucking Leeches From West Sumatra: A Discovery of Blue‐Eyed Litter Frog Leptobrachium waysapuntiense. *Ecology and Evolution*, *15*(10), e72235. https://doi.org/10.1002/ece3.72235

Several errors were identified in Figure 4, Table 3, and the final paragraph of the “Acknowledgments” section.

In Figure 4, the species‐level heatmap contains incorrect labels. Specifically, the “16Scp” and “12S” columns were mislabelled, and the column order does not match that of the family‐level heatmap on the right. In addition, the row labelled “Prionailurus planiceps” should read “Prionailurus bengalensis.” Despite these labeling inaccuracies, the numerical data within the heatmap remains correct. The corrected figure is shown below.

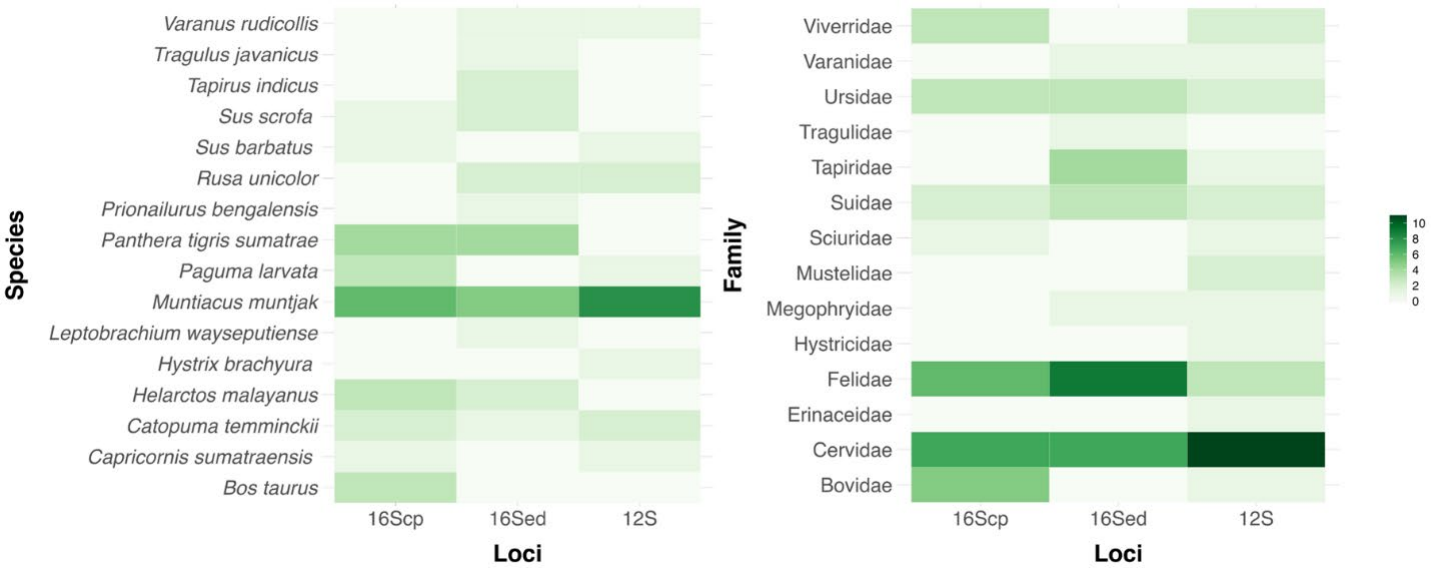

In Table 3, the row labelled “Panthera tigris sumatraensis” should be consistently corrected to “Panthera tigris sumatrae.”

In the “Acknowledgments” section, two minor errors were identified in the final paragraph regarding funding periods. The correct information is as follows: the funding period for JSPS KAKENHI (No. 23K05299) is 2023–2025 and for the Nagao Natural Environment Foundation, 2025.

We apologize for these errors.

