# Supplementary material for: Application of Invertebrate‐Derived DNA Barcoding (iDNA) in Blood Sucking Leeches From West Sumatra: A Discovery of Blue‐Eyed Litter Frog Leptobrachium waysapuntiense
Source: Ecol Evol. 2025 Oct 1;15(10):e72235. doi: 10.1002/ece3.72235 (PMC12486191; doi:10.1002/ece3.72235)
Supplement: Supplementary file 1 — Figure S1: ece372235‐sup‐0001‐FigureS1.docx. [file ECE3-15-e72235-s001.docx]

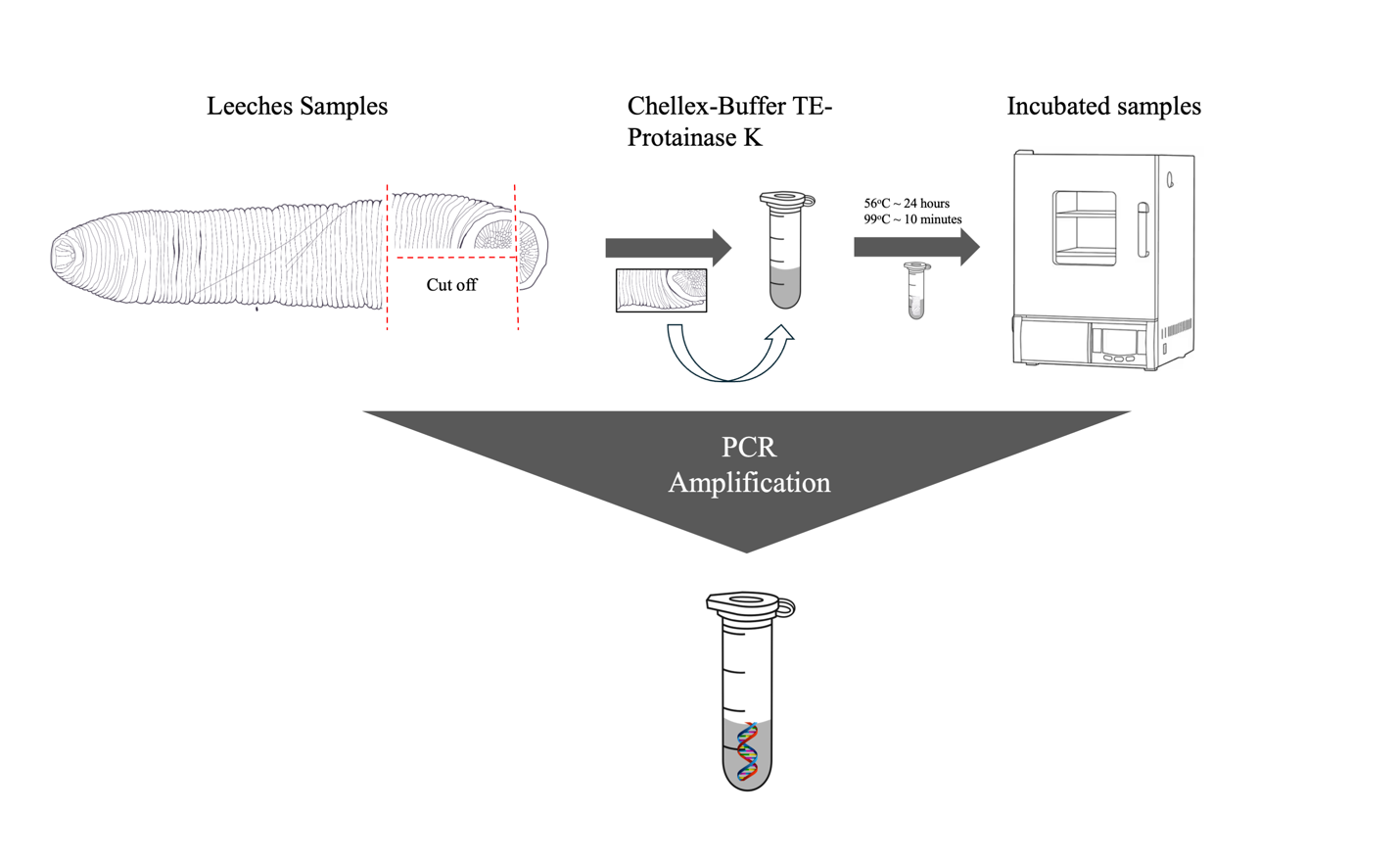
Supplementary

Figure 1. Schematic strategy of DNA extraction from individual leeches using the Chelex-TE method to obtain host DNA
